# Levetiracetam and N-Cadherin Antibody Alleviate Brain Pathology Without Reducing Early Epilepsy Development After Focal Non-convulsive Status Epilepticus in Rats

**DOI:** 10.3389/fneur.2021.630154

**Published:** 2021-02-24

**Authors:** Una Avdic, Matilda Ahl, My Andersson, Christine T. Ekdahl

**Affiliations:** ^1^Inflammation and Stem Cell Therapy Group, Division of Clinical Neurophysiology, Lund University, Lund, Sweden; ^2^Epilepsy Center, Department of Clinical Sciences, Lund University, Lund, Sweden

**Keywords:** synaptic proteins, seizures, epilepsy, antiepileptic drugs, levetiracetam, N-cadherin antibody, non-convulsive status epilepticus

## Abstract

Focal non-convulsive status epilepticus (fNCSE) is a neurological condition characterized by a prolonged seizure that may lead to the development of epilepsy. Emerging experimental evidence implicates neuronal death, microglial activation and alterations in the excitatory and inhibitory synaptic balance as key features in the pathophysiology following fNCSE. We have previously reported alterations in the excitatory adhesion molecule N-cadherin in rats with fNCSE originating from the hippocampus that subsequently also develop spontaneous seizures. In this study, fNCSE rats were treated intraperitoneally with the conventional anti-epileptic drug levetiracetam in combination with intraparenchymal infusion of N-cadherin antibodies (Ab) for 4 weeks post-fNCSE. The N-cadherin Ab was infused into the fornix and immunohistochemically N-cadherin Ab-stained neurons were detected within the dorsal hippocampal structures as well as in superjacent somatosensory cortex. Continuous levetiracetam treatment for 4 weeks post-fNCSE reduced microglia activation, including cell numbers and morphological changes, partly decreased neuronal cell loss, and excitatory post-synaptic scaffold protein PSD-95 expression in selective hippocampal structures. The additional treatment with N-cadherin Ab did not reverse neuronal loss, but moderately reduced microglial activation, and further reduced PSD-95 levels in the dentate hilus of the hippocampus. Despite the effects on brain pathology within the epileptic focus, neither monotherapy with systemic levetiracetam nor levetiracetam in combination with local N-cadherin Ab administration, reduced the amount of focal or focal evolving into bilateral convulsive seizures, seizure duration, or interictal epileptiform activity during 1 month of continuous electroenephalogram recordings within the hippocampus after fNCSE. Behavioral tests for spatial memory, anxiety, social interaction and anhedonia did not detect gross behavioral differences between fNCSE rats with or without treatment. The results reveal the refractory features of the present rodent model of temporal lobe epilepsy following fNCSE, which supports its clinical value for further therapeutic studies. We identify the persistent development of epilepsy following fNCSE, in spite of partly reduced brain pathology within the epileptic focus.

## Introduction

Status epilepticus can be a serious medical condition and life threatening if not interrupted. Convulsive features are typically observed in most patients, where seizures lasting for more than 30 min can be fatal and need immediate medical attention. However, 20–40% of all SE cases manifest with more subtle symptoms including altered consciousness, automatism, chewing, and minor motor movement, commonly referred to as non-convulsive status epilepticus (NCSE). When NCSE involves only parts of the brain it is called focal NCSE (fNCSE). The subtle presentations of fNCSE pose clinical challenges in terms of diagnosis and treatment. The pathophysiological events that follow can over time result in epilepsy development with recurrent spontaneous seizures.

Emerging evidence shows pathological features associated with fNCSE originating within the hippocampus (HPC), including neurodegeneration, neurogenesis, gliosis, excitatory-inhibitory imbalance, and molecular changes within the epileptic focus (1–5). Anti-epileptic drugs used for terminating seizure activity generally potentiate GABAergic tone and increase overall inhibitory signaling or aim at decreasing glutamate signaling by inhibiting NMDA receptors (6, 7). Other drugs, including levetiracetam, used widely in the clinic as an anti-convulsive/epileptic agent, target synaptic proteins such as synaptic vesicle protein 2A (SV2A), inhibit presynaptic calcium channels, and hence reduce general synaptic transmission in the brain (8–11). Current treatment recommendations for fNCSE include lorazepam/diazepam, midazolam, phenytoin, valproate, levetiracetam, or lacosamide if seizures continue (12).

Synaptic adhesion molecules are suggested to play an important role in maintaining the excitatory/inhibitory (E/I) balance, the latter postulated as a mechanism underlying the development of epilepsy (13). These structural synaptic components are involved in synaptic establishment, transmission and strength and are thus critical for fine-tuning the synaptic response (14, 15). Alterations in synaptic proteins are evident in both patients and in experimental models of SE and epilepsy (16–18). Recent experimental evidence showed altered synaptic protein expression in rodents following fNCSE, where synaptic proteins, found on both excitatory and inhibitory synapses, were altered within the epileptic focus (19). In particular, N-cadherin, an adhesion molecule involved in synapse development and maturation, and found primarily on excitatory synapses, was decreased following fNCSE specifically in rats that later developed spontaneous seizures. It is unknown whether or not the decreased expression may reflect a compensatory mechanism counteracting the seizure-induced hyperexcitability (19). Conditionally N-cadherin-ablated mice display reduced levels of post-synaptic proteins in glutamatergic excitatory synapses and reduced seizure severity in the kainic acid epilepsy model (20).

In the current study we aimed to modulate N-cadherin expression in rats that exhibited hippocampal fNCSE with intraparenchymal N-cadherin antibodies (Ab) in an attempt to further suppress N-cadherin and alter brain pathology within the epileptic focus and the subsequent development of spontaneous seizures during the first month after fNCSE. Behavior tests were performed for memory, anxiety and anhedonia. The fNCSE was interrupted with pentobarbital and we mimicked and evaluated the common clinical scenario of polypharmacy and therapy-resistance by administering the conventional anti-epileptic drug levetiracetam during the entire period post-fNCSE.

## Methods

### Animals

Adult male Sprague-Dawley rats (*n* = 36) weighing 200–250 g were procured from Charles River (Sulzfeld, Germany) and housed individually with a 12-h light/dark cycle and *ad libitum* food and water. Procedures were approved by the regional Malmö/Lund committee for experimental animal use and the Swedish board of Agriculture (protocol number M93-14). Animals were used in accordance with the European Community Council Directive (2010/63/EU) and the Swedish Animal Welfare Act (SFS 1988:534) and all experiments were performed in accordance with relevant guidelines and regulations. Every effort was made to limit the number of animals used. Rats were randomly divided into the different experimental groups and analyzed by researchers who were blind to the treatment condition.

### Group Assignment

**Figure 1:**
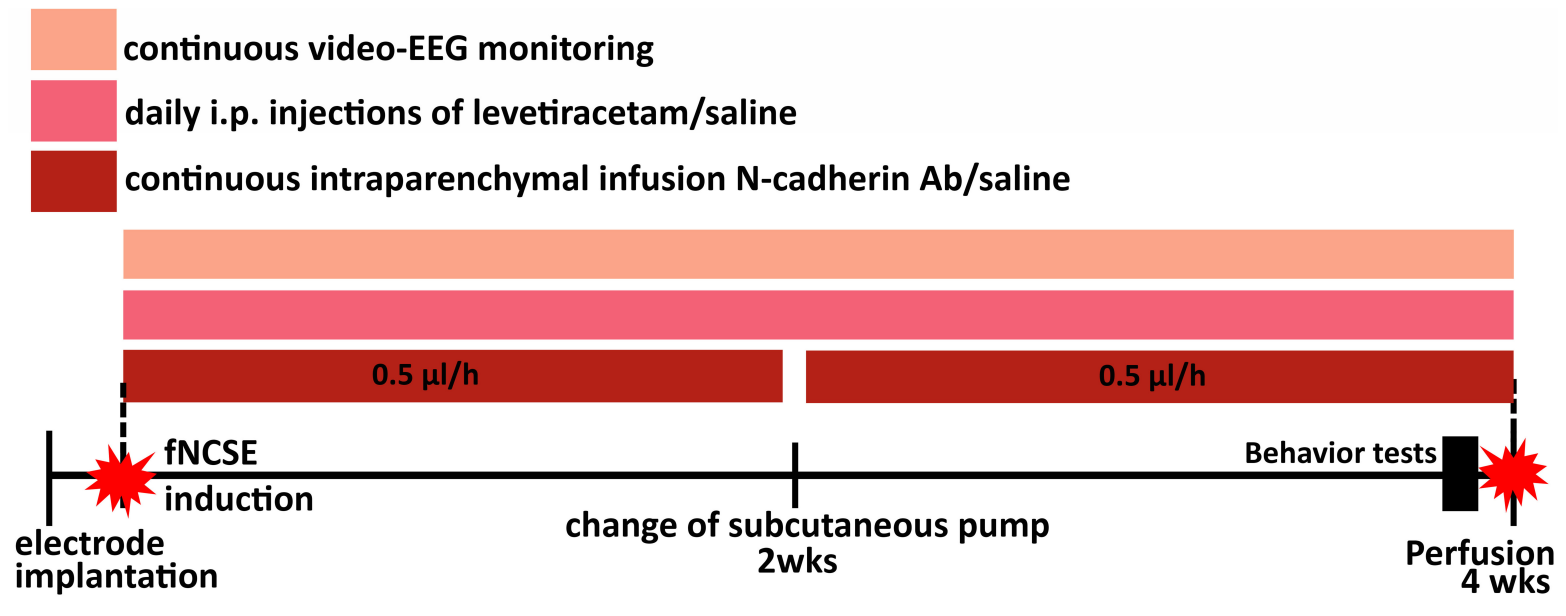
Schematic representation of the experimental set-up. Animals were divided into four groups; electrode- and canulae-implanted non-stimulated controls (Ctrls), focal non-convulsive status epilepticus (fNCSE)-Vehicle-Saline (Veh-Sal), fNCSE-Veh-Levetiracetam (Lev) and fNCSE-N-cadherin antibody (N-cad)-Lev. They were all implanted with electrodes and cannulas in the hippocampus (HPC) and after 1 w 3 groups were subjected to fNCSE and thereafter directly continuously infused (0.5 μl/h) with N-cadherin (1mg/ml) or saline through a subcutaneous pump and daily intraperitoneal injections (i.p.) of Levetiracetam (Keppra, 200mg/kg) or saline for 4 weeks. During three days prior to perfusion, all rats were subjected to behavior tests in the following sequential order: Open Field and Y-maze day 1, Cylinder test and Social Interaction test day 2, Porsolt test day 3. Sucrose Preference test continued simultaneously during day 1-3.

Rats were randomly divided into four groups: non-stimulated controls (Ctrl) that received intracerebral vehicle (phosphate buffer saline, PBS) and intraperitoneal (i.p.) saline injections (*n* = 10); rats with fNCSE that received either intracerebral vehicle and i.p. saline (*n* = 9) or i.p. levetiracetam (Keppra®, *n* = 6), and rats with fNCSE with intracerebral N-cadherin Ab and i.p. levetiracetam injections (*n* = 7, Figure 1). In addition, five naïve animals with low (0.5 mg/ml, *n* = 2) and high (1 mg/ml, *n* = 3) concentration of N-cadherin Ab were used for evaluation of antibody distribution, where the higher dose had a wider distribution and was selected for fNCSE rats.

### Surgeries and Electrically Induced Temporal Status Epilepticus

Animals were anesthetized with 2% isoflurane and implanted with a bipolar insulated stainless steel electrode (Plastics One, Roanoke, VA) into the right ventral CA1/CA3 region of the HPC [coordinates: 4.8 mm posterior and 5.2 mm lateral from bregma; and 6.3 mm ventral from dura, toothbar set at −3.3 mm, (21)] for stimulation and recording. A unipolar electrode was placed between the skull and adjacent muscle to serve as ground electrode. Additionally, a brain infusion cannula (Brain Infusion Kit 1, Alzet) was implanted in the fornix based on previous studies with similar technical approach (22, 23) to minimize surgery-induced trauma or immune response in the area of evaluation of seizure-induced pathology (coordinates: 1.0 mm posterior and 1.1 mm lateral to bregma; and 3.8 mm ventral to the flat skull position with bregma as reference) in the ipsilateral hemisphere. Following a week of recovery, rats were subjected to electrically induced fNCSE according to the protocol described in Jackson et al. (4). Electrode-implanted rats with no stimulations served as controls for the fNCSE rats. Initially, afterdischarge thresholds were determined for each rat by applying a square wave biphasic pulse (50 Hz) of 1 s train duration at a starting intensity of 10 μA, then increasing by 10 μA every 1 min until a 10 s afterdischarge was evoked. For inducing fNCSE, suprathreshold stimulation was applied for 1 h, with interruptions in the stimulation every 9th min to record electroencephalographic (EEG) activity for 1 min. Subsequently, the ictal EEG activity was recorded for another 2 h and the behavioral seizures were classified according to Racine's scale (24). There is a degree of heterogeneity in the pool of animals and susceptibility to seizures and seizure severity (2) and only rats that displayed self-sustained ictal electroencephalographic (EEG) activity for 2 h in the temporal lobe and mainly partial seizure semiology, for example, orofacial twitches, nodding, drooling, and unilateral forelimb clonus, according to Racine's scale, were included in this study (24). Behavioral symptoms and ictal EEG activity were completely interrupted, after 2 h of self-sustained fNCSE, by administration of pentobarbital (65 mg/kg).

### Intracerebral N-Cadherin Antibody Infusion and Intraperitoneal Levetiracetam Injections

All animals received brain infusion cannulas connected with osmotic pumps (2002, Alzet) carrying either mouse anti- N-cadherin Ab (1 mg/ml) (Abcam, UK, ab98952) or vehicle (PBS). Infusion rate was 0.5 μl/h for 2 weeks, or 1 w for naïve animals. Non-stimulated electrode-implanted rats were also connected with osmotic pumps carrying vehicle to serve as controls. The osmotic pumps were placed in the subcutaneous pocket in the dorsal region of the neck and replaced once after 2 weeks. Simultaneously, animals received daily i.p. injections of levetiracetam (200 mg/kg, (25) or saline for 4 weeks starting directly after fNCSE.

### Seizure and EEG Evaluations

Animals were continuously video-EEG–monitored (24 h/d) for 4 weeks throughout the experimental procedure (Powerlab and Lab- chart v8.1.1; AD Instruments, Dunedin, New Zealand; sampling frequency = 1,000 Hz). The EEG from intrahippocampal electrodes was visually evaluated and quantified in terms of EEG patterns during fNCSE, number of spontaneous and acute symptomatic seizures, and interictal activity (IA). Seizures [both acute symptomatic seizures within 1 week after fNCSE (26) (provoked symptoms), and spontaneous seizures starting day 8 post-fNCSE] (19) were defined as epileptiform EEG activity lasting ≥10 s with an evolving pattern, typically consisting of initial high-frequency low-amplitude activity that over time increases in amplitude and decreases in frequency as a spike–slow-wave pattern. Seizure frequency was quantified manually, and the total time animals exhibited seizure activity was divided into days 0–7 and 8–28 post-fNCSE. Seizure semiology was also evaluated during the course of 4 weeks of the experiment and divided into focal non-convulsive seizures or focal evolving into bilateral convulsive seizures (Racine scale) (24). IA was manually graded daily for 20 min according to a 0–5 scale (0 = none, 1 = <10 spikes/h, 2 = ~50 spikes/ h, 3 = ~ 80 spikes/h, 4 = ~ 100 spikes/h, and 5 = >150 spikes/h). Since IA increases in drowsiness and while falling to sleep, IA analyses were performed at approximately 7 am (when lights are turned on), a period characterized by sleepiness or sleeping rats.

### Behavior

At 4 weeks post-fNCSE, rats were subjected to behavior tests in order to evaluate memory, anxiety- and anhedonia-like behavior, and social interaction in the experimental groups. All tests were conducted during 3 days prior to perfusion, the same time of day and under normal room lighting. Two behavior tests were carried out every day (morning and afternoon) in the following sequential order: Open Field and Y-maze day 1, Cylinder test and Social Interaction test day 2, Porsolt test day 3. Sucrose Preference test continued simultaneously during day 1–3 before perfusion. Silence in the room was maintained for the duration of the tests. All analyses were quantified manually.

#### Open Field

The parameters in open field test spontaneous locomotor activity and anxiety-like behavior. The animals were placed individually in the center area of a square open field (80 cm × 80 cm), which was divided into 16 zones, 4 central and 12 peripheral. Animals were video recorded for 10 min and during this time locomotor activity was expressed as the number of squares crossed in the center and periphery of the open field. Anxiety-like behavior was evaluated by quantifying the time spent in each area. The open field chamber was washed with 95% ethanol solution between each rat (27, 28).

#### Y-Maze

Working memory was assessed in a Y-maze. A black Plexiglas Y-maze, elevated 100 cm from the floor, consisted of three arms with an angle of 120° between each arm. Each arm extended 50 cm with 10 cm width and 15 cm height. The three arms were randomly designated as A, B and C. Rats were placed individually in the center if the Y-maze and recorded for 10 min. The animals were allowed to freely explore the three arms, and should show a tendency to enter a less visited arm, as rodents typically prefer to investigate a new arm. The number of triads and number of entries into each arm was quantified. The parameters analyzed included the number of times visiting three different arms consecutively (e.g., ABC, BCA etc), that is, spontaneous alteration performance (SAP), the number of times the animal visited other arms and returned to the same arm (e.g., ABA, ACA), that is, alternative arm returns (AAR) and number of times the animal returned to the same arm (AAB, BAA etc.), that is, same arm returns (SAR) (29–31). These paradigms are based on the tendency of rats to explore new environments. Rats will alternate between arms and explore the least visited arm by using working memory. Thus, if working memory is impaired animals will score low on the measured parameters (29, 32, 33). The Y-maze was washed with 95% ethanol solution between each rat.

#### Cylinder Test

A previous study has reported motor deficits in rats with NCSE elicited with lithium pilocarpine (34). Thus, in order to evaluate potential motor deficits a cylinder test was performed to assess fore- and/or hindlimb lateralization in the rats. Rats were placed individually inside a glass cylinder (20 cm diameter × 70 cm tall). The rats were video recorded for 8 min and the number of times the rats touched the cylinder wall with the left and right forepaw respectively, was measured. The forelimb asymmetry ratio between the right and left paw was analyzed and plotted for each experimental group. The cylinder was washed with 95 % ethanol solution between each rat (35).

#### Porsolt Test

The porsolt swim test was performed to evaluate anhedonia in rats (36). The animals were placed in a glass cylinder (20 cm diameter × 70 cm tall) containing 25°C water (50 cm deep to prevent the rats tail from touching the bottom). Rats were recorded for 8 min and immobility, that is, the time the rats were not active other than that required to keep the rats head above water, was measured (36).

#### Social Interaction Test

Social interaction assesses the preference of rodents to engage in social behavior and overall social activity (37, 38). An intruder—a male, age-matched rat—was introduced into the animal home cage (425 × 276 × 153 mm) of individual animals. The intruder animals were unfamiliar with the experimental animal, with which they were paired for testing. Animals were recorded for 10 min, and the time and frequency of interaction (defined as contact, i.e., playing, sniffing of any part of the body of the intruder, following and chasing, initiated by the experimental rat) and passive (i.e., lack of interest when intruder initiates interaction) and active avoidance (i.e., actively avoiding when intruder initiates interaction) were quantified.

#### Sucrose Preference Test

Reduced sucrose preference suggests anhedonia in rats (39). Animals were deprived of water for 6 hrs and were afterwards presented with two bottles, one containing tap water and the other 2% sucrose water. The bottles were left in the cages throughout the experiment that lasted 72 h and daily switching position of bottles (left vs. right). The total intake of sucrose water and tap water was also analyzed in order to compare general fluid intake between experimental groups.

### Tissue Preparation

For immunohistochemistry, rats were transcardially-perfused with 0.9% saline and 4% paraformaldehyde (PFA). Brains were collected and post-fixed in PFA overnight, kept in 20% sucrose solution at 4°C for >24 h, cut in 30 μm coronal sections (10 series) on a freezing microtome, and stored in glycerol-based anti-freeze solution in −20°C.

### Immunohistochemistry

For immunohistochemistry, the following primary antibodies were used: rabbit anti-Iba1 (1:1,000, Wako, Japan, 019-19741), mouse anti-CD68/ED1 (1:200, AbD Serotec, Germany, MCA341R), mouse anti-PSD-95 (1:100, Abcam, UK, ab2723), goat anti – neuroligin 1 (NL-1) (1:200, Santa Cruz Biotechnology, USA, sc-365110), monoclonal mouse anti-gephyrin (1:3,000, Synaptic systems, Germany,147-111), goat anti-neuroligin 2 (NL-2) (1:200, Santa Cruz Biotechnology, sc-14087), mouse anti-NeuN (1:200, Abcam, ab104225) and polyclonal rabbit anti-doublecortin (DCX) (1:200, Synaptic systems, 326-003). Subsequently, free-floating sections were incubated with an appropriate primary antibody overnight at 4 °C in blocking serum. This was followed by 2 h of incubation in secondary antibody at room temperature. Intraparenchymal N-cadherin Ab diffusion was assessed by incubating free-floating sections with only secondary antibody. For each immunohistochemical assessment, some brain sections went through the entire protocol without primary antibody incubation, to serve as negative controls. The following secondary antibodies were used: Cy3- conjugated donkey anti-mouse/rabbit/goat (1:200; Jackson ImmunoResearch, UK, 715-165-150, 771-165-152, 705-165-147), Alexa-488 conjugated donkey anti-mouse/rabbit (1:200; Invitrogen, NY, USA, A32766, A32790) or Alexa-488 conjugated streptavidin (1:200, Invitrogen, Sweden, S11223). The sections were mounted on gelatin-coated slides and coverslipped using a glycerol-based mounting medium (DABCO, Sigma).

### Fluoro-Jade Staining

Sections were washed with potassium PBS (KPBS), hydrated, and pretreated with 0.06% potassium permanganate for 15 min, rinsed with distilled water, and treated with 0.001% Fluoro-Jade (Histo-Chem, Jefferson, AR, USA) for 30 min. They were subsequently washed with distilled water, dehydrated with ethanol and xylene, and coverslipped with PERTEX mounting medium.

### Cell Counts and Image Analysis

Quantifications of Iba1 and ED1-positive cells were performed ipsilaterally to the epileptic focus in the dentate hilus, granular cell layer (GCL), molecular layer (ML) of the dentate gyrus, CA1 and CA3 regions within 3–4 sections/animal (every 10^th^ section from −3.3 to −4.6mm posterior to bregma) using an Olympus BX61 epifluorescence microscope. The data is expressed as mean number of cells/section. For morphological analyses of microglia phenotypes in the same regions, a total number of 90–120 Iba1^+^ cells were analyzed per animal for three different subtypes: ramified (small soma and extensive dendritic tree), intermediate (larger soma and less extensive dendritic tree), and round/amoeboid (no processes), according to previously described definitions (22, 40). The relative occurrence of each subtype was expressed as the mean percentage of Iba1^+^ cells per section. DCX^+^ cell counting was performed in the subgranular zone (SGZ) / GCL ipsilaterally in 3–6 hippocampal sections and the data is expressed as mean number of DCX^+^ cells/section. Fluoro-Jade^+^ cell counting was performed in the ipsilateral dentate hilus, CA1, and CA3 in 3–6 hippocampal sections and the data is presented as mean number of Fluoro-Jade ^+^ cells/section. Number of NeuN^+^ cells was counted in ipsilateral CA1, CA3, GCL and dentate hilus by stereological measurements using a modified optical fractionator method, set to sample all cells above the first 10 μm from the surface of the section, with Olympus BH-2 microscope, 20× objective and Visiopharm software (Visiopharm, Denmark). Three sections were randomly selected from one of ten parallel series, located between 3.3 and 4.4 mm posterior to bregma. NeuN^+^ cells are presented as number of cells per mm^3^.

### Confocal Analysis

Intensity measurements of PSD-95, gephyrin, NL-1 and NL-2 expression in ipsilateral CA1, inner ML (iML) of the dentate gyrus, GCL (only gephyrin and NL-2), and the dentate hilus were performed with confocal laser scanning microscope (Zeiss, Germany), with a 561-nm excitation filter, ×63 oil-immersion objective, and ×5 zoom. Images were taken from 3 representative areas from each animal. Each image was acquired in a z-stack at an interval of 0.2 μm, on average 50 slices per z-stack. The images were analyzed in ImageJ software and the brightness and contrast corrected and noise reduced using the built-in ImageJ functions. Background intensity was measured in every image and subsequently subtracted from the mean gray value from each image in order to obtain a background-corrected mean gray value per animal.

To ensure lack of bias an observer blind to the treatment conditions conducted all analyses pertaining to cell counts and image analyses.

### Statistical Analysis

Statistical analyses were performed with non-parametric Mann-Whitney when comparing 2 groups, including grading of IA, using GraphPad Prism software (La Jolla, CA, USA). Comparisons between 3 groups were performed with Kruskal-Wallis statistical test, due to non-normal distribution evaluated by Shapiro Wilk normality test. Percentage of microglial morphology was normally distributed and analyzed with two-way analysis of variance (ANOVA) followed by Bonferroni *post hoc* test. Data are presented as median with upper quartile range, unless otherwise stated. Presence or lack of seizures following fNCSE with or without treatment was evaluated with chi-squared test. Differences were considered statistically significant at *P* ≤ 0.05.

## Results

### Distribution of N-Cadherin Antibody After Intraparenchymal Brain Infusion

**Figure 2:**
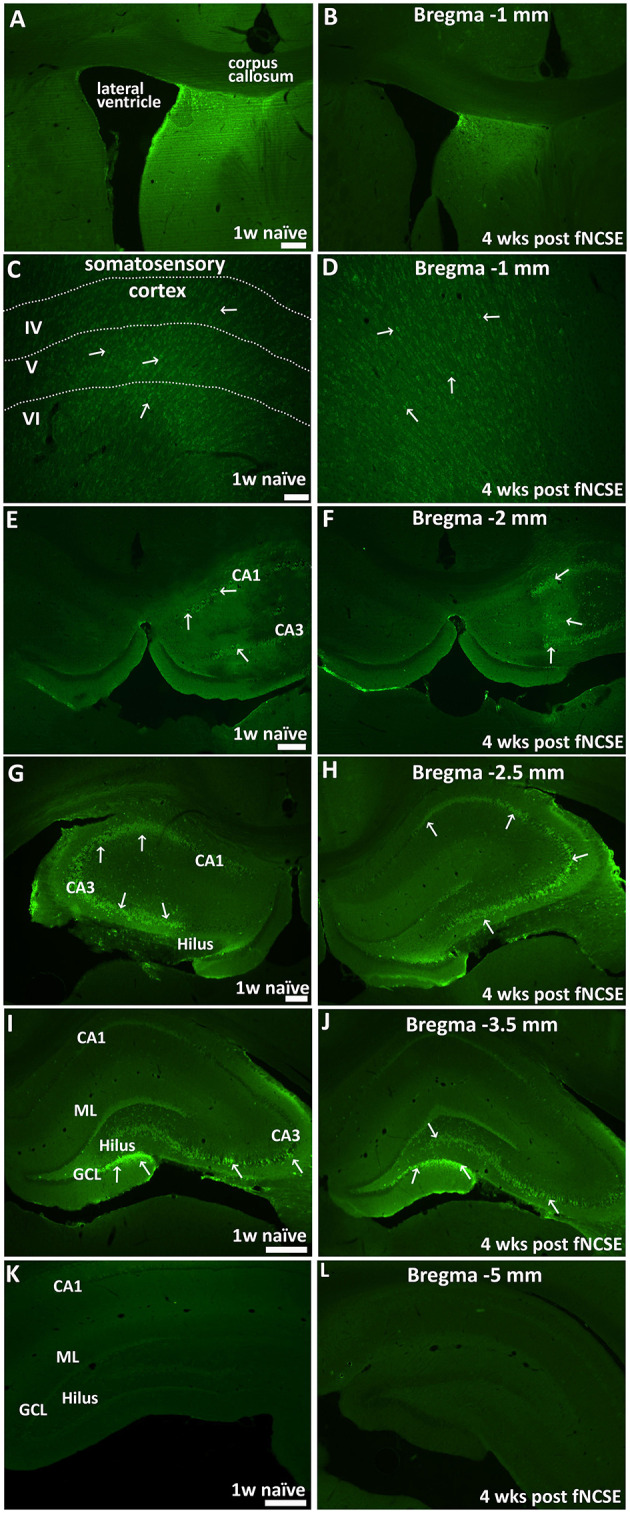
Distribution of intracerebral N-cadherin antibody 1 week post-infusion in naïve animals and 4 weeks following focal non-convulsive status epilepticus. Unilateral infusion of N-cadherin antibody (Ab) (1mg/ml), shows N-cadherin Ab –labeled cells **(A,B)** close to the infusion site and in proximity to the fornix, **(C,D)** in somatosensory cortex adjacent to infusion site, **(E-L)** in dorsal HPC, at physiological conditions 1 w post-infusion in naïve rats (*n* = 3) (left panel) and 4 weeks post-fNCSE and infusion (*n* = 7) (right panel). Arrows indicate strongly stained N-cadherin expressing cells. Scale bar is 100 μm in **(A–D, G,H)**, 500 μm in **(E,F,I**, and **L)**.

Unilateral infusion of N-cadherin Ab (1mg/ml) in the brain was first evaluated in a cohort of naïve rats (n = 3) to assess the presence and spread of the Ab in the brain. N-cadherin Ab expression was assessed by a secondary Ab targeting the N-cadherin Ab. The injection site in fNCSE rats treated with 1 mg/ml N-cadherin Ab was defined in the majority of animals by N-cadherin positive cells in a local, confined area above the injection site in close proximity to the fornix (*n* = 6 of 7, representative images in Figures 2A,B). We observed a continuous N-cadherin immunoreactivity proximal to the injection site, sparse staining in the supradjacent somatosensory cortex (Figures 2C,D) and a prominent increased immune reactivity within the dorsal hippocampus (Figures 2E–J, 1–3.5 mm post-bregma). The infusion of Ab did not seem to simply reach the ventral hippocampus as no increased immune reactivity was detected in the ventral hippocampus of any animals (Figures 2K,L), with the exception of one fNCSE animal. This animal showed immune reactivity as far as 5 mm post-bregma but did not differ compared to the other fNCSE animals in terms of seizure burden or other performed analyses.

### Seizure Semiology and EEG Evaluations During Focal Non-convulsive Status Epilepticus

**Figure 3:**
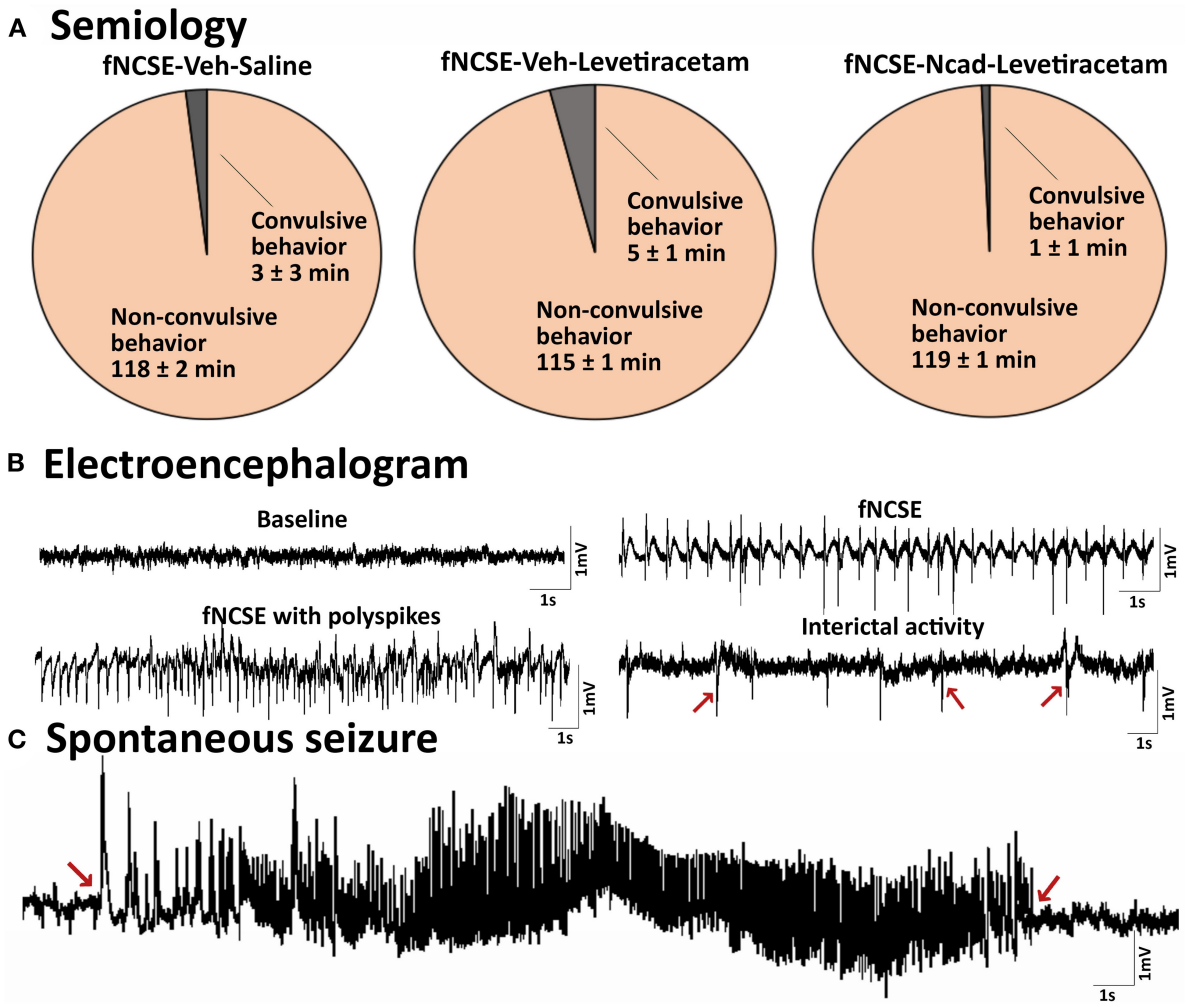
Seizure semiology and electroencephalogram in rats with focal non-convulsive status epilepticus **(A)** Pie charts showing mean percentage of time rats exhibit focal non-convulsive seizure semiology during 2 hours of self-sustained fNCSE in fNCSE-Veh-Sal, fNCSE-Veh-Lev and fNCSE-N-cad-Lev rats. **(B)** Representative baseline activity before stimulation, epileptiform activity during self-sustained fNCSE (1-3Hz), fNCSE activity with polyspiking, interictal epileptiform activity (indicated by arrows), **(C)** an example of spontaneous seizure with an evolving seizure pattern. Arrows mark the beginning and end of the seizure.

As a certain degree of heterogeneity is to be expected in susceptibility to seizures and seizure severity in any group of animals (2), we characterized and compared seizure semiology between groups to ascertain that animals irrespective of assigned treatment group displayed a homogenous profile prior to treatment. This was confirmed as the semiology during fNCSE was primarily and to the same extent non-convulsive in all three experimental groups (98% in fNCSE-Veh-Saline (Sal), 96% in fNCSE-Veh-Levetiracetam (Lev) and 99% of the time in fNCSE-N-cadherin (N-cad)-Lev, with <4% convulsive semiology with bilateral fore-/hindlimb clonus, Figure 3A). All groups showed similar EEG patterns during fNCSE, which consisted of a rhythmic low frequency spike-and-wave activity pattern (1–3 Hz), interrupted with periodic higher frequencies of rhythmic discharges, including occasional polyspiking (Figure 3B), similar to what can be found in clinical practice.

### No Alterations in Seizure Burden or Interictal Epileptiform Activity After 1 Month of Treatment With Levetiracetam and N-Cadherin Antibody Following fNCSE

To further align our study to clinical practice, seizures post-fNCSE were divided in two groups (acute symptomatic or spontaneous seizures) depending on time elapsed from fNCSE. Clinical seizures occurring within the first week after a systemic insult or brain injury are defined as possibly injury-provoked or acute symptomatic seizures (26), which may not always lead to the development of epilepsy. Seizures occurring the following weeks are classified as spontaneous un-provoked seizures associated with epilepsy. The majority of rats experienced EEG and video-confirmed acute symptomatic seizures during the first week following fNCSE (56% in fNCSE-Veh-Sal, 80% in fNCSE-Veh-Lev and 57% in fNCSE-N-cad-Lev), suggesting that N-cadherin and levetiracetam treatments were not sufficient to decrease the number of possible provoked seizures following the initial insult. Similarly, the latency to acute symptomatic seizures did not differ between the experimental groups (fNCSE-Veh-Sal 3 h *u* = 68.6 vs. fNCSE-Veh-Lev 1 h *u* = 5.49 vs. fNCSE-N-cad-Lev 1 h *u* = 130). In total 70, 50, and 88% of animals with acute symptomatic seizures developed subsequent spontaneous seizures during week 2–4 in the fNCSE-Veh-Sal, fNCSE-Veh-Lev, fNCSE-N-cad-Lev group, respectively. In total, most rats developed spontaneous seizures during the following 3 weeks post-fNCSE (fNCSE-Veh-Sal 67%, fNCSE-Veh-Lev 83%, fNCSE-N-cad-Lev 100%, during week 2–4), with or without previous acute symptomatic seizures, and the latency to the first spontaneous seizure after week 1 did not differ between the animals (fNCSE-Veh-Sal 277.5 h *u* = 445.1 vs. fNCSE-Veh-Lev 240 h *u* = 493.5 vs. fNCSE-N-cad-Lev 247 h *u* = 313), suggesting that the treatments did not postpone the onset of the development of epilepsy.

**Figure 4:**
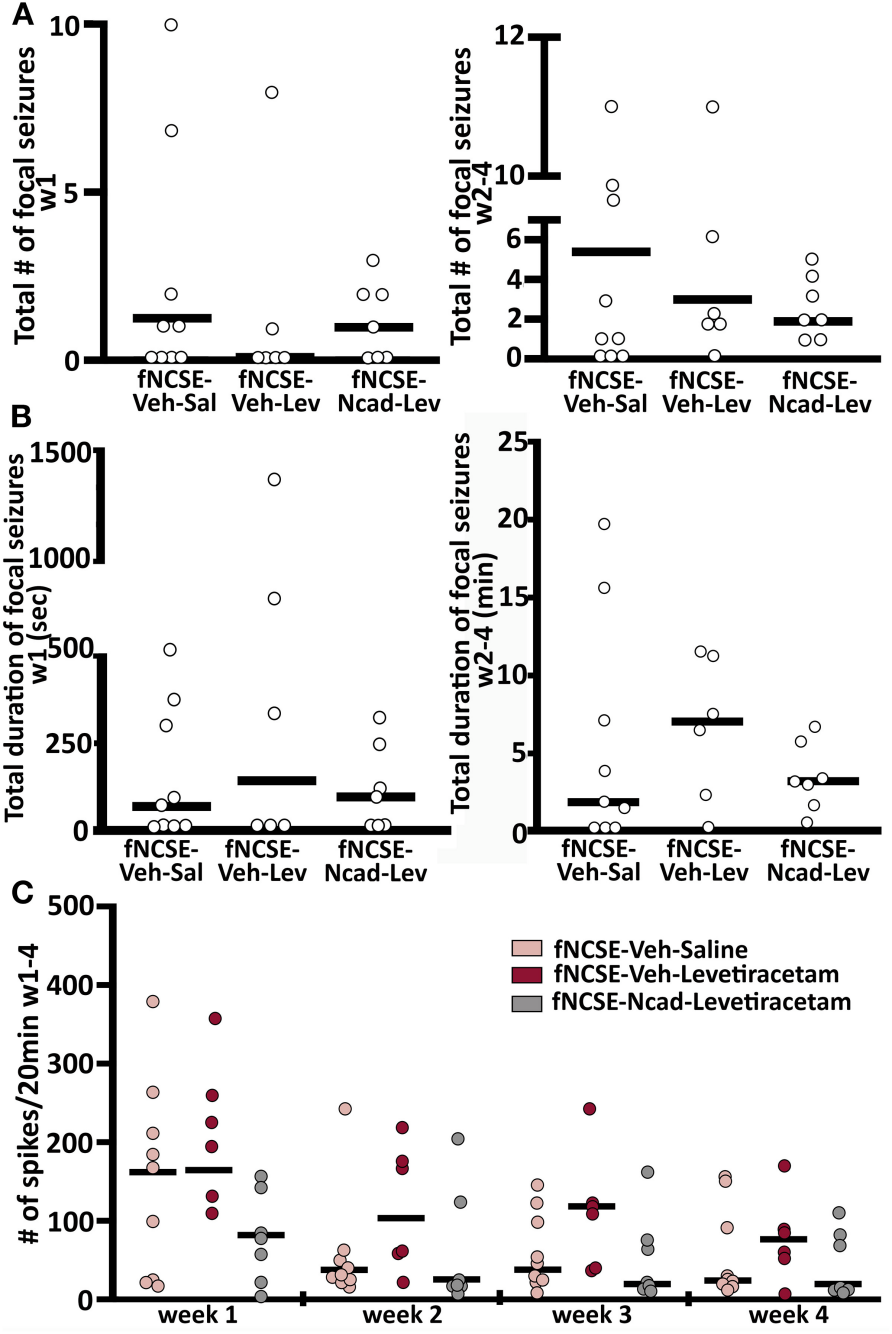
Seizure burden and interictal activity following focal non-convulsive status epilepticus **(A)** Total number and **(B)** total duration (min) of focal non-convulsive seizures during week 1 and week 2-4 post-fNCSE, in fNCSE-Veh-Sal, fNCSE-Veh-Lev and fNCSE-Ncad-Lev groups, respectively. **(C)** Grading of the amount of interictal epileptiform activity (IA) per 20 min of EEG recordings. Data are presented as median ± individual numbers; fNCSE-Veh-Sal, n = 9; fNCSE-Veh-Lev n = 6; fNCSE-N-cad-Lev n = 7. ^*^P ≤ 0.05.

The majority of seizures in animals that developed epilepsy were categorized as focal/non-convulsive seizures with altered consciousness and ambulatory or masticatory (oro-fascial twitches/chewing) behavior, but all three groups displayed occasional focal seizures evolving into bilateral convulsive seizures. The risk of this secondary generalization was not affected by the treatment (total number of focal seizures evolving into bilateral convulsive seizures week 1 and week 2–4 in fNCSE-Veh-Sal 0 *u* = 0 and 0.5 *u* = 11.14, fNCSE-Veh-Lev 0 *u* = 0 and 0 *u* = 1.8, and fNCSE-Ncad-Lev 0 *u* = 2.6 and 0 *u* = 12.8, respectively, total duration of focal seizures evolving into bilateral convulsive seizures week 1 and week 2–4 in fNCSE-Veh-Sal 0 *u* = 0 s and 2.0 *u* = 11.2 min, fNCSE-Veh-Lev 0 *u* = 0 s and 7.1 *u* = 11.5 min, and fNCSE-Ncad-Lev 0 *u* = 132 s and 3.33 *u* = 5.6 min, respectively). Moreover, neither total number of focal seizures nor total duration of focal seizures differed between treatment groups during week 1 and 2-4 (Figures 4A,B), and there were no group differences in numbers of animals with or without seizures during week 1 and week 2–4 post fNCSE (*p* > 0.05 with chi-squared test). Overall, the treatment did not reduce total number of either focal or focal evolving into bilateral convulsive seizures that developed over the entire 4 week period post-fNCSE (number of focal seizures: fNCSE-Veh-Sal 5.50 *u* = 9.67 vs. fNCSE-Veh-Lev 4.00 *u* = 9.46 vs. fNCSE-N-cad-Lev 3.00 *u* = 8.00, focal evolving into bilateral convulsive seizures: fNCSE-Veh-Sal 0.50 *u* = 10.6 vs. fNCSE-Veh-Lev 0 *u* = 1.71 vs. fNCSE-N-cad-Lev 0 *u* = 13.2). Thus, treatment with N-cadherin Ab in combination with levetiracetam or levetiracetam alone did not alter the severity of the seizures. Furthermore, we sub-analyzed the seizure severity, evaluated total number and total duration of seizures on a weekly basis during week 2, 3, and 4 in all three experimental groups and found no differences over time (data not shown). Finally, the grading of IA was similar in all three experimental groups during the four weeks post-fNCSE and quantification of spikes did not reveal any changes between the groups at week 1–4 (Figure 4C).

### No Gross Changes in Cognitive Test Outcome After 1 Month of Treatment With Levetiracetam and N-Cadherin Antibody Following fNCSE

Seizures and epilepsy development are frequently associated with cognitive alterations. We aimed to assess functional consequences on cognition and behavioral traits following fNCSE with and without treatment. Analysis of anxiety-related behavior conducted in an open field did not reveal any changes in the time spent in the center of the open field in untreated rats with fNCSE compared to Ctrls (Supplementary Table 1.1), nor could we observe changes in the treatment groups (Supplementary Figure 1A). Furthermore, evaluation of the numbers of squares crossed in the box was not changed in the experimental groups (Supplementary Table 1.2 and Figure 1A), suggesting unaltered locomotor activity and lack of motor impairments. Anhedonia, also associated with hippocampal networks, was assessed with Porsolt test. The duration of immobility did not differ between untreated rats with fNCSE and Ctrls (Supplementary Table 1.3), or rats treated with N-cadherin Ab and/or levetiracetam (Supplementary Figure 1B). This was further confirmed when assessing anhedonia by a sucrose preference test, which showed no alterations in sucrose intake between the untreated fNCSE and Ctrls (Supplementary Table 1.4) or fNCSE untreated vs. the two treated groups, though the values differed between the two treatment groups, Supplementary Figure 1C. Subsequent analysis of working memory, a hippocampal-dependent task, performed in a Y-maze, did not reveal any alterations in SAP, AAR or SAR in untreated animals with fNCSE compared to Ctrls Supplementary Table 1.5) or to rats treated with N-cadherin Ab and/or levetiracetam (Supplementary Figure 1D). Consistent with the open field results, the total number of arm entries did not differ between the experimental groups (Supplementary Table 1.5 and Figure 1D). Evaluation of social behavior in animals with fNCSE showed a significant increase in the duration of contacts made in a social interaction test in untreated fNCSE animals compared to Ctrls (Supplementary Table 1.6), but no differences were observed following treatment with levetiracetam or levetiracetam in combination with N-cadherin Ab (Supplementary Figure 1E). Other parameters for social interaction such as the number of contacts made, and passive and active avoidance were also evaluated but did not reveal any changes in rats with fNCSE compared to Ctrl (Supplementary Tables 1.7, 1.8, and 1.9) or after treatment with N-cadherin Ab and/or levetiracetam (Supplementary Figure 1E). Moreover, no changes in lateralization of left or right paw was detected in untreated fNCSE animals compared to controls (Supplementary Table 1.10) or to any of the treated experimental groups (Supplementary Figure 1F). Sub-analysis of the performance in the behavioral tests exhibited by rats that developed spontaneous seizures week 2–4 after fNCSE (67, 83, and 100% in the fNCSE-Veh-Sal, fNCSE-Veh-Lev, fNCSE-N-cad-Lev group, respectively) did not show any additional changes between groups (data not shown).

### Altered Brain Pathology After 1 Month of Treatment With Levetiracetam and N-Cadherin Antibody Following fNCSE

**Figure 5:**
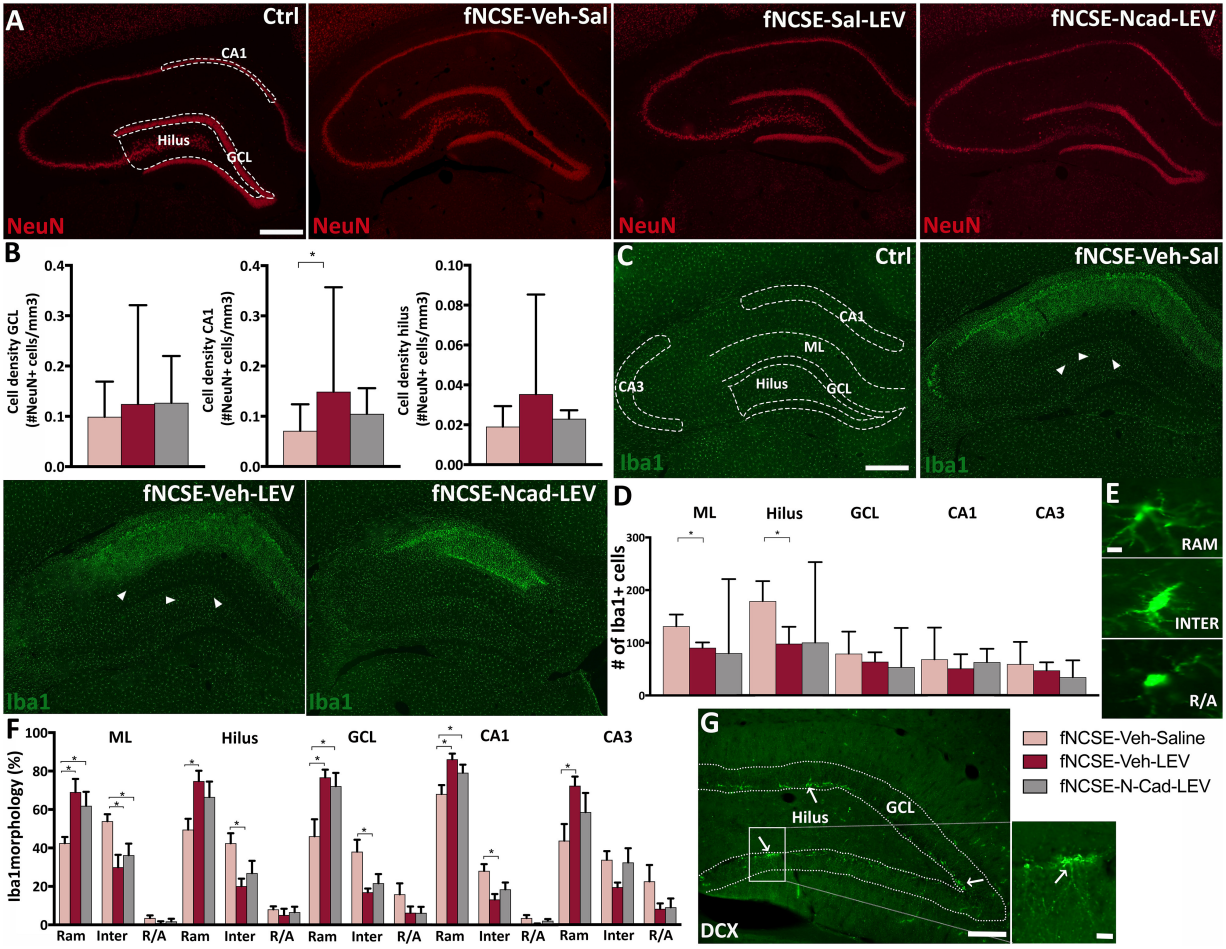
Altered neuronal loss and microglial activation following 4 weeks of levetiracetam and N-cadherin antibody treatment post- focal non-convulsive status epilepticus. **(A)** Representative images of NeuN^+^ cells in Ctrls, fNCSE-Veh-Sal, fNCSE-Veh-Lev, fNCSE-N-cad-Lev and **(B)** stereological quantification in GCL, CA1 and dentate hilus. **(C)** Representative images of Iba1^+^ cells (arrow heads), **(D)** quantification of Iba1^+^ cells in the HPC, **(E)** different morphological phenotypes of microglia, **(F)** relative percentage of Iba1 morphology in different sub-regions of HPC, **(G)** DCX^+^ cells (arrows) in the subgranular zone of the hippocampal dentate gyrus. Data are presented as median ± range, except Iba1 morphological phenotypes which is presented as mean ± SEM; fNCSE-Veh-Sal, n = 6–9; fNCSE-Veh-Lev n = 6; fNCSE-N-cad-Lev n = 7. ^*^P ≤ 0.05. ML = molecular layer. RAM = ramified, INTER = intermediate, R/A = round/amoeboid Iba1. Scalebar = 500 μm in **(A and C)**, 10 μm in **(E)** and 250 μm in **(G)** and 20 μm in inset in **(G)**.

We further explored whether N-cadherin Ab and levetiracetam treatment would affect common pathophysiological changes associated with fNCSE (19) including neuronal cell death and inflammation. Stereological evaluations of subregions of the HPC did not reveal changes in numbers of NeuN^+^ cells in the GCL of the HPC in untreated rats with fNCSE compared to Ctrl (Supplementary Table 1.11) or following levetiracetam with or without N-cadherin Ab infusion (Figures 5A,B). In the CA1 region, however, fNCSE had significantly less NeuN^+^ cells than Ctrls (Supplementary Table 1.11). This decrease was partly reversed by levetiracetam treatment but was not significant in the group receiving levetiracetam in combination with N-cadherin Ab infusion (Figures 5A,B). A decrease in NeuN^+^ cells was also observed in the dentate hilus following fNCSE compared to Ctrl (Supplementary Table 1.11), but not affected by treatment (Figure 5B). No changes were detected in area volume during stereological analysis in either treatment group (data not shown).

Fluoro-Jade staining was performed at 4 weeks post-fNCSE to assess cell damage. Analysis showed significantly more Fluoro-Jade^+^ cells in sub-regions of the epileptic focus compared to Ctrl (Supplementary Table 1.12), without any differences in fNCSE rats treated with levetiracetam with or without N-cadherin Ab infusion compared to untreated fNCSE (dentate hilus: fNCSE-Veh-Sal 0.25 *u* = 0.56 vs. fNCSE-Veh-Lev 0 *u* = 0.19 vs. fNCSE-N-cad-Lev 0.25 *u* = 0.48), CA1 (fNCSE-Veh-Sal 2.86 *u* = 4.00 vs. fNCSE-Veh-Lev 2 *u* = 3.60 vs. fNCSE-N-cad-Lev 0 *u* = 2.62) or CA3 (fNCSE-Veh-Sal 1.75 *u* = 2.59 vs. fNCSE-Veh-Lev 0.75 *u* = 2.53 vs. fNCSE-N-cad-Lev 0.75 *u* = 1.79).

Quantification of Iba1^+^ microglia cells within the epileptic focus in sub-regions of the HPC showed a pronounced increase in the ML and dentate hilus in fNCSE rats compared to Ctrls (Supplementary Table 1.13). Levetiracetam treatment reduced the number of Iba1^+^ cells in these sub-regions, but not significantly in combination with N-cadherin Ab infusion (Figures 5C,D). Further immunohistochemical evaluations in the CA1 region showed a similar pattern with increased number of Iba1^+^ cells following fNCSE compared to Ctrls Supplementary Table 1.13). Numbers were, however, not decreased by levetiracetam or by N-cadherin Ab and levetiracetam (Figures 5C,D). No changes were detected in Iba1^+^ cells in the CA3 and GCL, in any of the groups (Supplementary Table 1.13) and Figures 5C,D). Furthermore, number of Iba1^+^/ED1^+^ cells were upregulated in the ML, CA1, and CA3 of fNCSE rats compared to controls (Supplementary Table 1.14), but with no further differences following treatment with levetiracetam or levetiracetam in combination with N-cadherin Ab (ML: fNCSE-Veh-Sal 7.67 *u* = 19.9 vs. fNCSE-Veh-Lev 3.83 *u* = 5.93 vs. fNCSE-N-cad-Lev 3.00 *u* = 25.3, CA1: fNCSE-Veh-Sal 41.0 *u* = 78.8 vs. fNCSE-Veh-Lev 22.2 *u* = 33.4 vs. fNCSE-N-cad-Lev 13.0 *u* = 42.4, CA3: fNCSE-Veh-Sal 32.3 *u* = 45.6 vs. fNCSE-Veh-Lev 8.67 *u* = 23.2 vs. fNCSE-N-cad-Lev 10.0 *u* = 21.9).

When comparing the morphological profiles of activated microglia we observed a significant interaction between ramified and intermediate phenotypes in untreated fNCSE rats compared to Ctrls in ML, hilus, CA1 and CA3 (Supplementary Tables 1.15, 1.16, 1.17, and 1.18), with a decreased ramified and an increased intermediate profile following fNCSE. However, rats treated with levetiracetam and N-cadherin in combination with levetiracetam, displayed an increased ramified and decreased intermediate profile compared to untreated fNCSE rats in the ML. Levetiracetam alone induced similar phenotypic changes in the dentate hilus, GCL, CA1 and CA3, and levetiracetam in combination with N-cadherin Ab infusion in the GCL and CA1 (Figures 5E,F). Sub-analysis of NeuN, Iba1, and Iba1/ED1 results from rats that developed spontaneous seizures week 2–4 after fNCSE (67, 83, and 100% in the fNCSE-Veh-Sal, fNCSE-Veh-Lev, fNCSE-N-cad-Lev group, respectively) did not show any additional changes between groups (data not shown).

Quantification of DCX^+^ cells was performed in the GCL/SGZ (Figure 5G) and analysis showed no changes in untreated fNCSE rats compared to Ctrls (Supplementary Table 1.19), or compared to fNCSE with or without treatment (fNCSE-Veh-Sal 252 *u* = 505 vs. fNCSE-Veh-Lev 207 *u* = 383 vs. fNCSE-N-cad-Lev 377 *u* = 507). However, analysis showed that DCX levels in the fNCSE groups positively correlated to the total number of spontaneous seizures week 2–4 (*r* = 0.62, *p* = 0.012), as well as the number of focal evolving into bilateral convulsive seizures (*r* = 0.54, *p* = 0.03), which partly explains the high variation in number of DCX^+^ cells within the fNCSE groups.

### Reduction in Postsynaptic Excitatory Protein PSD-95 in the Dentate Gyrus Following Treatment With N-Cadherin Ab Post-fNCSE

**Figure 6:**
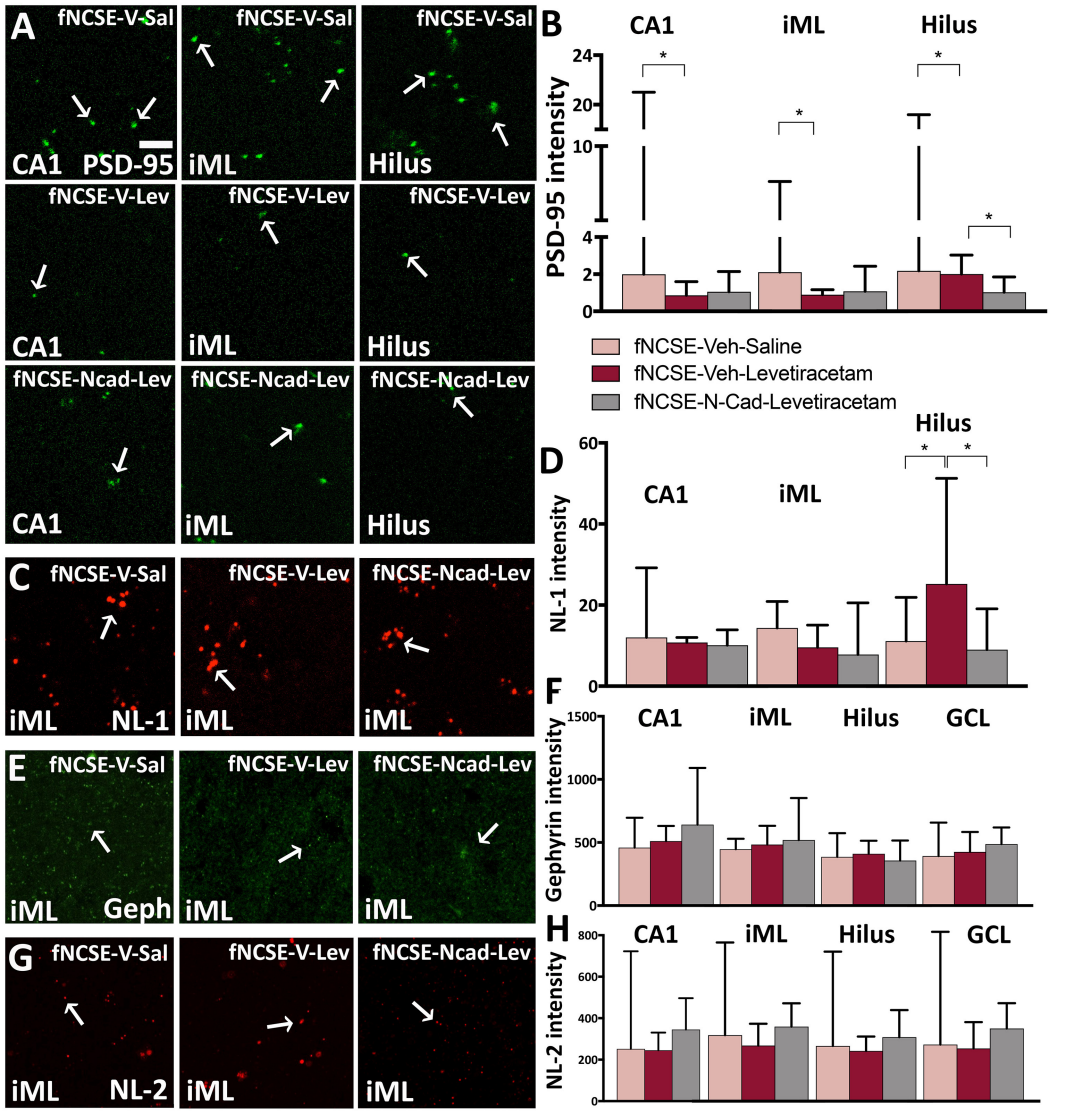
Reduced PSD-95 expression following 4 weeks of levetiracetam and N-cadherin antibody treatment post- focal non-convulsive status epilepticus. Photomicrographs of **(A)** PSD-95 clusters and **(B)** quantification of PSD-95 intensity, **(C,D)** photomicrographs and quantification of neuroligin-1 (NL-1), **(E,F)** gephyrin and **(G,H)** NL-2 clusters. Arrows in **(A)**, **(C)**, **(E)**, and **(G)** point toward clusters of synaptic proteins. Data are presented as median ± range; fNCSE-Veh-Sal, n = 6–9; fNCSE-Veh-Lev n = 6; fNCSE-N-cad-Lev *n* = 7. ^*^P ≤ 0.05. iML = inner molecular layer. Scalebar in **(A)** = 5 μm for all photomicrographs.

We have previously reported altered expression of excitatory and inhibitory synaptic proteins in the epileptic focus following fNCSE (19). In line with this, we saw an increased expression of excitatory-related scaffolding protein PSD-95 in fNCSE rats compared to Ctrl (Supplementary Table 1.20) in the CA1 region of HPC. This increase was reversed by levetiracetam treatment alone, but not with N-cadherin Ab and levetiracetam (Figures 6A,B). Within the iML, an area where the hippocampal granule cells mainly receive inhibitory afferents from interneurons in addition to excitatory input, and where we have previously shown seizure-related changes (4, 41), we observed an increased expression of PSD-95 clusters in fNCSE compared to Ctrls (Supplementary Table 1.20). This increase was reversed by levetiracetam, but again did not reach significance with levetiracetam in combination with N-cadherin Ab (Figure 6B). In the dentate hilus, PSD-95 expression was not altered in animals following fNCSE compared to Ctrls (Supplementary Table 1.20). There was, however, a small decrease in PSD-95 expression in rats with N-cadherin Ab infusion and levetiracetam compared to untreated fNCSE rats and rats treated with levetiracetam (Figure 6B).

No changes were detected in excitatory synaptic adhesion molecule NL-1 expression in the CA1 or iML following fNCSE (Supplementary Table 1.21), nor were there alterations following treatment with levetiracetam and N-cadherin Ab, or levetiracetam alone (Figures 6C,D). In the dentate hilus, the untreated fNCSE group was not different compared to Ctrls (Supplementary Table 1.21), but NL-1 levels were increased in levetiracetam-treated fNCSE rats compared to untreated fNCSE rats as well as fNCSE rats with levetiracetam and N-cadherin Ab infusion (Figure 6D).

Analysis of the inhibitory scaffolding synaptic protein gephyrin in the iML showed increased expression in untreated animals following fNCSE compared to Ctrls (Supplementary Table 1.22), but levels were not reversed after treatment with levetiracetam or N-cadherin Ab combined with levetiracetam (Figures 6E,F). In the GCL, levels of gephyrin were also increased in untreated fNCSE rats compared to Ctrls (Supplementary Table 1.22), but no changes compared to treatment groups (Figure 6F). In CA1 and hilus no changes in gephyrin expression were detected following fNCSE (Supplementary Table 1.22) and expression was not altered in animals following treatment (Figure 6F).

Further analysis of the inhibitory synaptic adhesion molecule NL-2 did not reveal any changes in expression levels in the iML, CA1, hilus, and GCL following fNCSE compared to control group (Supplementary Table 1.23), nor was there a difference after the two treatments (Figures 6G,H). Sub-analysis of PSD-95, NL-1, gephyrin, and NL-2 levels from rats that developed spontaneous seizures week 2–4 after fNCSE (67, 83, and 100% in the fNCSE-Veh-Sal, fNCSE-Veh-Lev, fNCSE-N-cad-Lev group, respectively) did not show any additional changes between groups (data not shown).

## Discussion

A fNCSE poses clinical challenges for proper diagnosis, prognosis and treatment. In the current study, we describe how brain pathology in a model of fNCSE in rodents may be altered by the commonly used anti-convulsive drug levetiracetam in systemic monotherapy and levetiracetam in combination with a local intracerebral infusion of an Ab against the excitatory synaptic adhesion molecule N-cadherin within the epileptic focus. Microglial activation, neurodegeneration, and the excitatory synapse-associated PSD-95 expression were modulated, however, the subsequent development of spontaneous seizures and amount of interictal epileptiform activity were not counteracted after 4 weeks of either treatment post-fNCSE.

We have recently reported a decreased expression of N-cadherin within the hyperexcitable epileptic focus at 4 weeks following fNCSE. This reduction was specifically observed in fNCSE rats with subsequent development of spontaneous seizures and not in rats with only acute symptomatic seizures within 1 w post-fNCSE (19). In the present study, where we administered N-cadherin Ab locally within the epileptic focus in addition to levetiracetam in an attempt to further decrease hyperexcitability within the epileptic focus, we did reduce the expression of excitatory postsynaptic scaffolding protein PSD-95, decreased microglia activation and neuronal death, but without a detectable difference in the development of subsequent spontaneous seizures. Cell death within the dentate hilus and CA1 of the HPC are prominent pathological features associated with epilepsy (42) and previous studies have shown neuroprotective effects of levetiracetam in both epileptic and non-epileptic disorders, including pilocarpine-induced convulsive SE in rodents (8, 43, 44), stroke and traumatic brain injury (45–48). The present combination of local administration of N-cadherin Ab and systemic levetiracetam treatment reduced PSD-95 expression in the dentate hilus, whereas levetiracetam alone decreased PSD-95 expression also in the iML. PSD-95 is known to modulate synaptic transmission (49, 50) and conditional ablation of N-cadherin at excitatory synapses has been shown to decrease post-synaptic excitatory scaffolding proteins such as PSD-95 and increase clusters of inhibitory post-synaptic scaffold proteins such as gephyrin, suggesting N-cadherin as a seizure-promoting protein (20). In addition, long-term blocking of N-cadherin leads to synapse elimination and loss of post-synaptic PSD-95 expression, supporting a possible role for N-cadherin in PSD-95 regulation as well as synaptic transmission (51). Thus, the combinatorial treatment-induced decrease in PSD-95 expression observed in the present study may reflect decreased hyperexcitability within the epileptic focus.

Impaired cognitive functions and increased risk of mood disorders are commonly associated with epilepsy (52–55). An experimental model of NCSE, induced by administration of low-dose pilocarpine, has similar behavioral deficits (34). In the present electrical model of fNCSE, we could not confirm gross behavioral changes, either as working memory deficiency, anhedonia or increased anxiety with the performed behavioral tests at 4 weeks post-fNCSE, with or without treatment. However, it is possible that behavioral deficits arise at later stages when the epileptic environment has been more established. Here we merely state that treatment with levetiracetam and N-cadherin Ab did not induce detectable negative effects on the cognitive performances in rats at 4 weeks post-fNCSE.

None of the present treatments altered the number of acute symptomatic or spontaneous seizures following fNCSE, which is in contrast to previous reports on reduced number of generalized tonic-clonic seizures during 2 weeks of levetiracetam treatment after pilocarpine-induced convulsive SE (56), even if video/EEG-recordings in that specific case were performed <24 h per day. The discrepancy can be attributed to either dose-dependent treatment effects, rat strain differences, or, perhaps more likely, the difference in the SE model and subsequent features of the spontaneous seizures. Indeed, differences in SE models have previously shown discrepancies in terms of the anti-convulsive effects observed with levetiracetam. Studies showed that the anti-convulsive effect was completely absent in the maximal electroshock seizure (MES) and pentylenetetrazol (PTZ) models (57), whereas in amygdala kindled rats the same treatment showed potent protective effects against seizures (58). However, despite early contradicting experimental observations, levetiracetam is today widely used in the clinic and is known to have anti-convulsive properties in patients with epilepsy. The known underlying mechanisms by which levetiracetam facilitates these mechanisms are mainly through inhibition of the synaptic vesicle protein 2A (45) and by reducing seizure-induced changes in synaptic remodeling genes and thus decreasing seizure propensity (59). Whether or not levetiracetam may be more efficient in reducing severe generalized tonic-clonic seizures compared to focal seizures is yet to be confirmed (60). In the present model of fNCSE we did not detect any differences between the effect of levetiracetam on focal non-convulsive and focal evolving into bilateral convulsive seizures. The additional local treatment with N-cadherin infusion within the epileptic focus had no further effect on seizure development. The Ab-exposed region within the epileptic focus may have been too small to have an impact or the alterations in N-cadherin and PSD-95 expression were not large enough to change seizure susceptibility. In addition, we cannot exclude the possibility of a gradual decrease in N-cadherin Ab function during the 2 week-infusion period or levetiracetam masking potential effects of N-cadherin Ab on seizure-induced pathology, although the two treatments have different mechanisms of action and primarily target pre- and post-synaptic compartments, respectively. However, it is also plausible that a reduced seizure burden (anti-convulsive effect) may manifest at later stages after 4 weeks post-fNCSE, which could be considered in future studies of the disease-modifying effects of levetiracetam and N-cadherin Ab treatment of refractory epilepsy.

## Conclusion

We conclude from the current study that fNCSE leads to pathophysiological changes within the epileptic focus in the brain that to some extent can be reversed within 1 month by daily monotherapy with levetiracetam. Only a minor additional change was confirmed with the addition of intracerebral N-cadherin Ab infusion within the epileptic focus. Despite the effects on brain pathology, the treatment did not alter the subsequent development (frequency, severity and duration) of spontaneous seizures or the amount of interictal activity. The current experimental model of electrically-induced fNCSE mimics clinical fNCSE and we report for the first time the development of therapy-resistant temporal lobe seizures in this model. It makes it a promising model for future evaluations of therapeutic strategies for refractory epilepsy.

## Data Availability Statement

The original contributions generated for the study are included in the article/Supplementary Material, further inquiries can be directed to the corresponding author/s.

## Ethics Statement

The animal study was reviewed and approved by the regional Malmö/Lund committee for experimental animal use and the Swedish board of Agriculture (protocol number M93-14).

## Author Contributions

CE and UA: study concept, drafting of the manuscript, study supervision, and critical revision. CE, UA, MAh, and MAn: data acquisition, data analysis, and interpretation. UA and MAh: animals and surgery. All authors critically reviewed and approved the manuscript.

## Conflict of Interest

The authors declare that the research was conducted in the absence of any commercial or financial relationships that could be construed as a potential conflict of interest.
