# Supplementary material for: Levetiracetam and N-Cadherin Antibody Alleviate Brain Pathology Without Reducing Early Epilepsy Development After Focal Non-convulsive Status Epilepticus in Rats
Source: Front Neurol. 2021 Feb 24;12:630154. doi: 10.3389/fneur.2021.630154 (PMC7943745; doi:10.3389/fneur.2021.630154)
Supplement: Supplementary file 1 [file Data_Sheet_1.docx]

Supplementary Material

|  |  | | |
| --- | --- | --- | --- |
|  | **Test** | **Ctrl** | **fNCSE-Veh-Saline** |
| Behavior tests | | | |
| 1.1 | OF Time in centre (sec) | 7 u=10.8 | 8 u=13.1 |
| 1.2 | OF squares crossed (no) | 53 u=64.8 | 70 u=120 |
| 1.3 | Porsolt immobility (sec) | 2 u=4.36 | 3 u=8.10 |
| 1.4 | Sucrose intake (ml) | 138.5 u=236.4 | 161.5 u=218.7 |
| 1.5 | Y-maze (no) | SAP: 20 u=22.3 | 18.5 u=30.5 |
|  |  | AAR: 8 u=10.4 | 10.5 u=23.3 |
|  |  | SAR: 0 u=1.00 | 0.5 u=1.10 |
|  |  | Arm entries: 29.5 u=33.6 | 33 u=50.6 |
| 1.6 | SI duration (sec/min) | **29.6 u=38.1** | **39.3 u=44.9*** |
| 1.7 | SI contact (no/min) | 2.5 u=2.82 | 2.67 u=3.22 |
| 1.8 | Passive avoidance (no/min) | 0 u=0.11 | 0 u=0.24 |
| 1.9 | Active avoidance (no/min) | 0 u=0.37 | 0 u=0.29 |
| 1.10 | Cylinder test (no of touches) | Left: 3.60 u= 6.81 | 4 u=5.10 |
|  |  | Right: 3.40 u=6.86 | 4 u=9.30 |
| Immune response, neuronal death and regeneration | | | |
| 1.11 | NeuN**+** cells (cells/mm^3^) | GCL: 0.144 u=0.179 | 0.099 u=0.149 |
|  |  | **CA1: 0.143 u=0.155** | **0.071 u=0.097*** |
|  |  | **Hilus: 0.055 u=0.059** | **0.019 u=0.026*** |
| 1.12 | Fluoro-Jade+ cells (no) | **Hilus: 0 u=0** | **0.25 u=0.56*** |
|  |  | **CA1: 0 u=0** | **2.86 u=4.00*** |
|  |  | **CA3: 0 u=0** | **1.75 u=2.59*** |
| 1.13 | Iba1+ microglia cells (no) | **ML: 90.8 u=124** | **131 u=145*** |
|  |  | **Hilus: 103 u=134** | **179 u=205*** |
|  |  | **CA1: 43.5 u=55.4** | **68.0 u=115.8*** |
|  |  | CA3: 43.8 u=67.2 | 59.3 u=86.3 |
|  |  | GCL: 70.2 u=95.9 | 79.0 u=103 |
| 1.14 | Iba1+/ED1+ cells (no) | **ML: 2.13 u=14.3** | **7.67 u=19.9*** |
|  |  | **CA1: 1.67 u=10.0** | **41.0 u=78.8*** |
|  |  | **CA3: 2.71 u=18.3** | **32.3 u=45.6*** |
| 1.15 | Iba1 morphology ML (%) | **RAM: 63.2 ± 5.06** | **41.1 ± 3.37*** |
|  |  | **INTER: 34.2 ± 4.76** | **55.3 ± 3.93*** |
|  |  | R/A: 2.62 ± 0.96 | 3.59 ± 1.61 |
| 1.16 | Iba1 morphology Hilus (%) | **RAM: 67.8 ± 4.84** | **49.5 ± 5.63*** |
|  |  | **INTER: 28.5 ± 3.81** | **42.5 ± 5.22*** |
|  |  | R/A: 3.67 ± 1.50 | 8.03 ± 1.58 |
| 1.17 | Iba1 morphology CA1(%) | **RAM: 76.0 ± 5.08** | **43.7 ±8.59*** |
|  |  | **INTER: 20.8 ± 3.96** | **33.7 ± 4.49*** |
|  |  | R/A: 3.19 ± 1.30 | 22.5 ± 8.51) |
| 1.18 | Iba1 morphology CA3(%) | **RAM: 75.6 ± 6.01** | **46.1 ±8.80*** |
|  |  | **INTER: 21.2 ± 4.54** | **38.0 ± 6.24*** |
|  |  | R/A: 3.17 ± 1.60 | 15.9 ± 5.73 |
| 1.19 | DCX+ cells in GCL/SGZ (no) | 215.4 u=253.9 | 252 u=505 |
| Synaptic proteins | | | |
| 1.20 | PSD-95 intensity | **CA1: 0.99 u=1.04** | **1.96 u=4.15*** |
|  |  | **iML: 0.95 u=1.20** | **2.10 u=3.61*** |
|  |  | Hilus: 1.69 u=2.22 | 2.04 u=3.07 |
| 1.21 | NL-1 intensity | CA1: 10.0 u=11.8 | 12 u=23.4 |
|  |  | iML: 10.2 u=13.2 | 14.4 u=19.8 |
|  |  | Hilus: 20.7 u=34.6 | 11.1 u=17.8 |
| 1.22 | Gephyrin intensity | **iML: 312 u=360** | **446 u=514*** |
|  |  | **GCL: 292 u=368** | **392 u=502*** |
|  |  | CA1: 403 u=451 | 460 u=586 |
|  |  | Hilus: 256 u=325 | 386 u=461 |
| 1.23 | NL-2 intensity | iML: 200.0 u=299 | 264 u=498 |
|  |  | CA1: 238 u=281 | 252 u=538 |
|  |  | Hilus: 188 u=239 | 221 u=454 |
|  |  | GCL: 170 u=249 | 227 u=493 |

**Supplementary Table 1.** **Statistical analysis** **of behavior tests, histopathology and synaptic protein expression in focal non-convulsive status epilepticus (fNCSE) rats compared to electrode- and cannula-implanted non-stimulated controls (Ctrls)**. OF=open field, SI= social interaction, SAP= spontaneous alteration performance, SAR= spontaneous arm return, AAR= alternative arm return, GCL= granular cell layer, iML = inner molecular layer, RAM = ramified, INTER = intermediate, R/A = round/ameboid, No = number. Data are presented as median± range, except data presenting Iba1 morphology which is presented with mean ± SEM, Ctrl: n= 10, fNCSE-Veh-Sal: n = 6-9. *P ≤ 0.05


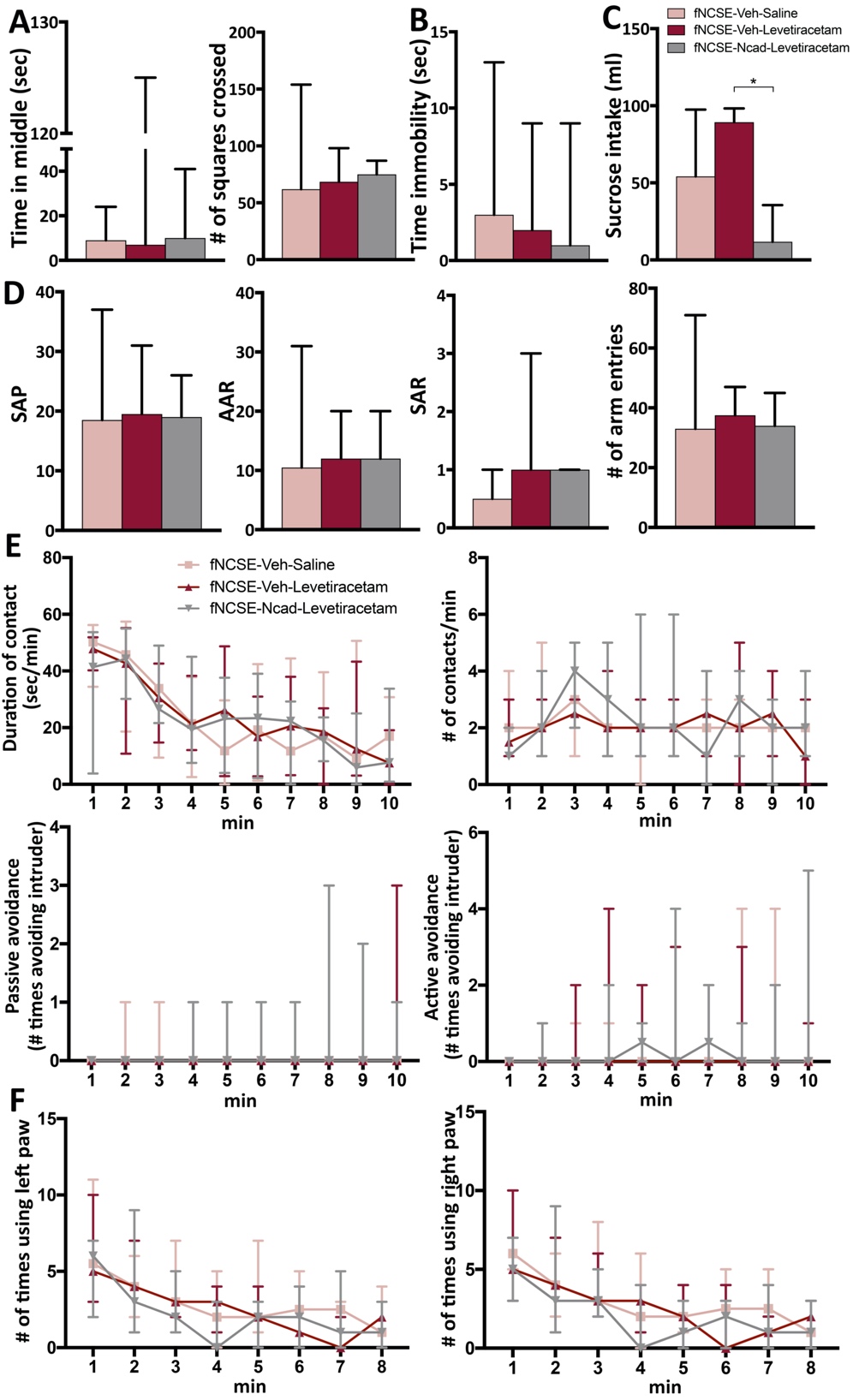


**Supplementary Figure 1.** **Locomotion, anhedonia anxiety-related behavior, and social interaction in rats 4 weeks following focal non-convulsive status epilepticus. (A)** Time spent in the central area and the number of squares crossed in Open Field, **(B)** total time spent immobile in water in Porsolt test, and **(C)** the amount of sucrose intake in the Sucrose Preference test. **(D)** Spatial working memory assessed with Y-maze where spontaneous alteration performance (SAP), alternative arm returns (AAR), same arm returns (SAR) and the total number of arm entries are quantified in fNCSE-Veh-Sal, fNCSE-Veh-Lev and fNCSE-N-cad-Lev. **(E)** Quantification of social interaction parameters as time spent interacting, number of contacts initiated by the resident rat, passive and active avoidance, and **(F)** number of times the left and right forepaw, respectively, are used in Cylinder test. Data are presented as median± range; fNCSE-Veh-Sal, n = 6-9; fNCSE-Veh-Lev n=6; fNCSE-N-cad-Lev n=7. *P ≤ .05.
